# Metabolome and transcriptome analysis reveal the pigments biosynthesis pathways in different color fruit peels of *Clausena lansium* L. *Skeels*

**DOI:** 10.3389/fpls.2024.1496504

**Published:** 2025-01-31

**Authors:** Zhichang Zhao, Mark Owusu Adjei, Ruixiong Luo, Huaping Yu, Yali Pang, Jian Wang, Yu Zhang, Jun Ma, Aiping Gao

**Affiliations:** ^1^ Tropical Crops Genetic Resources Institute Chinese Academy of Tropical Agricultural Sciences, Haikou, Hainan, China; ^2^ Landscape Architecture College of Sichuan Agricultural University, Chengdu, Sichuan, China; ^3^ Guangxi Subtropical Crops Research Institute, Nanning, Guangxi, China

**Keywords:** *Clausena lansium* L. *Skeels*, fruits peels, anthocyanins, flavonoids, fruit peel color

## Abstract

**Introduction:**

The color of *Clausena lansium* L. *Skeels* cv. *Jixin* fruit peel is brown (BP), while the mutant cv. *Zijin* had purple fruit peels (PP). The coloration of the peels was attributed to significant differences in chlorophyll, carotenoid, and anthocyanin content between BP and PP.

**Methods:**

This study investigates the biosynthetic metabolic activities in the brown and purple peels of *Clausena lansium* L. *Skeels* using metabolomics and transcriptomics. It aims to identify metabolic pathways and differentially expressed genes related to flavonoids and anthocyanins biosynthesis.

**Results:**

The PP (purple peel) has higher levels of a-carotene and b-carotene but lower levels of chlorophyll a, chlorophyll b, and lutein compared to BP. Zeaxanthin was absent from both peels, suggesting that the b-carotene hydroxylase enzyme is not active. Both peels contain delphinidin-based (Dp) and cyanidin-based (Cy) anthocyanins, but not pelargonidin-based (Pg). The total anthocyanin content and the Dp/Cy ratio are higher in PP than in BP. The delphinidin, cyanidin, and mallow derivatives in the PP were significantly higher than in the BP. The increase of total anthocyanin content and Dp/Cy ratio may be the main reason for the peel color changing from brown to purple. The significant increase of F3H expression in purple peels suggested a higher efficiency of catalyzing the conversion of naringenin into dihydroflavonols in the PP, leading to the higher content of total anthocyanin. Despite the significant increase of FLS expression in PP, the contents of kaempferol, quercetin, and myricetin significantly decreased, suggesting that the increase of FLS expression did not lead to an increase in flavonol biosynthesis.

**Discussion:**

The competition between F3’H and F3’5’H may determine the ratio of Dp/Cy, the higher levels of F3’H, F3’5’H, and UFGT expression, lead to the increase accumulation of total anthocyanin and Dp/Cy in PP. The deficiency of Pg in both peels resulted from the substrate specificity of the DFR enzyme. The research also describes the transition in color from BP to PP and details of the biosynthetic pathways for carotenoids and anthocyanins, elucidating the molecular processes underlying anthocyanin production.

## 1 Introduction

*Clausena lansium* L. *Skeels* is one of the Rutaceae families that originated in China (Zhaoa et al., 2004; Chang et al., 2018). Studies have revealed that all parts of the plant are useful, including leaves, seeds, roots, and fruits, which contain significant amounts of antioxidant and anti-inflammatory properties (Arscott and Tanumihardjo, 2010; Chang et al., 2018). The fruit has a pleasant flavor, with a tough, thin, flexible peel that is greasy and sticky. Similar to the fruits, literature has revealed that the peel is a potential source of natural antioxidants (Liu et al., 2019). Accordingly, the peel tissues play important roles in affecting fruit development and quality, contributing to the whole plant’s usefulness and growth (Pongprasert et al., 2011). However, understanding of the composition and biosynthesis pathways in *Clausena lansium* L. *Skeels* fruit is limited. Different fruit colors are controlled by phytochemicals (Lopes et al., 2016). The phytochemicals are classified as primary and secondary metabolites based on their functional metabolites. Chlorophyll, carotenoids, and flavonoids, as secondary metabolites, play important roles in the coloration of fruits and flowers (Guerriero et al., 2018; Owusu Adjei et al., 2021; Elshafie et al., 2023). Flavonoids are classified into flavonols, flavones, isoflavones, and anthocyanins according to their structure and modifications (Li et al., 2021). The flavonoids perform many functions (Sun et al., 2022), like regulating cell growth, attracting pollinators and insects, and protecting against biotic and abiotic stresses (Dias et al., 2021). In flavonoid biosynthesis, structural genes like Phenylalanine ammonia-lyase (PAL), Cinnamate 4-hydroxylase (C4H), 4-coumarate-coa ligase (4CL), Chalcone synthase (CHS), and Chalcone isomerase (CHI) are responsible for the generation of naringenin, which is the primary precursor of specific flavonoids (Li et al., 2010; Qiu et al., 2013). Anthocyanins are members of the flavonoid group of phytochemicals, synthesized in the cytosol and then glycosylated and acylated to form various anthocyanin derivatives, stored in the vacuoles (Zeng et al., 2024; Prasad et al., 2010; Luo et al., 2022). The flavonoid 3′-hydroxylase (F3’H), flavonoid 3′,5′-hydroxylase (F3’5’H), Dihydroflavonol-4-reductase (DFR), Anthocyanin synthetase (ANS), Flavonoid 3-O-glucosyltransferas (UFGT), and Glutathione S-transferase (GST) are important for the biosynthesis of anthocyanin, and the activity of F3’H, F3’5’H, and DFR are important for the ration of Dp/Cy (Ren et al., 2022). F3′H and F3′5′H are key enzymes in the flavonoid biosynthesis pathway. They are responsible for controlling hydroxylation at the 3′ and 5′ of reddish-purple and blue pigments (Brugliera et al., 2013). The diversity of anthocyanin molecules is related to branching the pathway to alternative ways in which dihydroflavonols can be transformed either with the help of F3′H or F3′5′H. Similarly, the DFRs play a crucial role in the biosynthesis of anthocyanins (Peng et al., 2020). They convert DHK (dihydrokaempferol), DHQ (dihydroquercetin), or DHM (dihydromyricetin) into leucoanthocyanidins, which are then converted into colored anthocyanidins by the ANS (Li et al., 2010; Qiu et al., 2013). Some plant species, such as *Camellia sinensis* and *Petunia* (Li et al., 2022), DFRs have strict substrate specificity, which leads to some lacking pelargonidin-based anthocyanins (Pg) (Chen et al., 2020). Next, anthocyanidins are further decorated by methylation, acylation, and glycosylation (Alseekh et al., 2020). Most of the studies have been conducted in model plants, often with respect to the production of flavonoid-based pigments (Chen et al., 2022). Usually, biosynthesis regulated by transcription factors that form the MBW ternary complexes consisting of MYBs, bHLHs, and WDs (Sun et al., 2022). The objective of the present study is to integrate metabolomics and transcriptomic analyses to reveal the biosynthesis pathways that control the peel pigments and discover the key genes that determine the biosynthesis of anthocyanin and the branching formation of the pathways in *Clausena lansium* L. *Skeels*.

## 2 Materials and methods

### 2.1 Plant materials

The ‘*Jixin*’ brown fruit and ‘*Zijin*’ purple fruit were collected from the Wampee Resources Nursery Institute of Fruit. The brown and purple peels were separated carefully from the fruits, frozen in liquid nitrogen immediately, and then transferred to −80°C until used.

### 2.2 RNA sequencing

Wuhan MetWare Biotechnology performed the transcriptome sequencing (Li et al., 2021). Total RNA was isolated using RNAprep Pure Plant Plus (DP441, TIANGEN, Beijing, China) and enriched for total mRNA using poly-T oligo-attached magnetic beads. The NEBNext1 Ultra RNA Library Prep Kit (NEB, Ipswich, MA, USA) was used for library construction. mRNA was fragmented, synthesized first-strand cDNA using a random hexamer primer and M-MuLV reverse transcriptase, and then synthesized second-strand cDNA. The 30 ends of the cDNA fragments were methylated and then ligated to adaptors for hybridization. Purification was performed using AMPure XP beads (Beckman Coulter, Beverly, MA, USA). After adding the index (X) primer, universal PCR primers, and high-fidelity DNA polymerase, PCR performed. The PCR products were purified and assessed by the Agilent Bioanalyzer 2100 system. The sequencing was performed on the Illumina HiSeq2500TM (Illumina, San Diego, CA, USA). The entire experiment was repeated three times (Li et al., 2021).

### 2.3 Heat map, GO and KEGG annotation

The heat map was constructed using Euclidian distances and complete linkage grouping with the heat map package in R (www.r-project.org), and the relative quantitative values of metabolites were normalized, transformed, and clustered through agglomerate hierarchical clustering. Metabolite correlation was assessed using the Pearson Correlation Coefficient and Cytoscape software (www.cytoscape.org). To further identify alternative metabolic pathways, differential metabolites were subjected to grouping and enrichment of metabolic pathways using MetaboAnalyst 4.0 software (www.metaboanalyst.ca), GO, and KEGG databases. The identified differential metabolites reacted to biochemical pathways according to the labeling in KEGG (http://www.kegg.jp/pathway).

### 2.4 Metabolic analysis

All the metabolites and soluble contents analysis were performed by High Performance Liquid Chromatography(HPLC) in a Thermo Ultimate 3,000 system equipped with an ACQUITY UPLC HSS T3 (150 × 2.1 mm, 1.8 µm, Waters) column at 40°C. The temperature of the autosampler was set at 8°C. Gradient elution of analytes was carried out with 0.1% formic acid in water (C) and 0.1% formic acid in acetonitrile (D) or 5 mmol/L ammonium formate in water (A), and acetonitrile (B) at a flow rate of 0.25 ml/min. Injection of 2 μL of each sample was done after equilibration. An increasing linear gradient of solvent B (v/v) was applied as follows: 0–1 min, 2%B/D; 1–9 min, 2%–50% B/D; 9–12 min, 50%–98% B/D; 12–13.5 min, 98% B/D; 13.5–14 min, 98%–2% B/D; 14–20 min, and 2% D-positive model (14–17 min, 2% B-negative model). Metabolic pathway enrichment and topological analysis were performed using the MetPA database (www.metaboanalyst.ca) to analyze metabolic pathways related between the two peels. The screening of significantly different metabolites was followed by variable importance in projection (VIP) ≥ 1 and fold change ≥2 or ≤0.5.

### 2.5 Anthocyanin content detection

For the measurement of the anthocyanin content of each cultivar, 0.25g of the sample was ground into fine powder in liquid N2 and then homogenized in 1 ml of anthocyanin extracts [methanol: distilled water: methane acid:trifluoroacetic acid (70:27:2:1, v/v/v/v)] at 4°C for 24 h. After samples were centrifuged, the supernatant were filtered using medium-speed filter paper, and the filtrate was the passed through a 0.22-μm reinforced nylon membrane filter before subjecting it to high performance liquid chromatography (HPLC) analysis.

### 2.6 Carotenoid content detection

Carotenoids were extracted following the method described by Liu et al. with slight modifications (Zhou et al., 2007). Carotenoids were determined on a reverse phase Analytical YMC Carotenoid Column C30 (150 × 4.6 mm i.d., 3 μm, Wilmington, NC, USA) using a Waters HPLC system with a photodiode array detector. Operation was conducted under subdued light to avoid carotenoid degradation. Identification of carotenoids was performed by comparison with standard spectra. Quantification was performed using the calibration curve generated with commercially available β-carotene, α-carotene, zeaxanthin, and lutein in standards (Sigma-Aldrich).

### 2.7 Chlorophyll content detection

Chlorophyll (Chl) content was measured following the procedure described by Mark 2022 (Owusu Adjei et al., 2022). The fruits peels were collected. First, peels tissues were ground using liquid nitrogen. Chl was then extracted with 80% (v/v) acetone for an hour under low light intensity. Extraction was carried out several times during the reverse centrifugal tube to accelerate the process. Second, the samples were centrifuged at 12000 g for 10 min, and then the clear liquid was collected to determine Chl content. Finally, Chl content was determined by spectrophotometry. The experiments were performed thrice using independent biological replicates.

### 2.8 Quantitative real-time PCR analysis

To validate the RNA-Seq results, 10 gene involved in flavonoid biosynthesis were selected for further validation using qRT-PCR and their relative expression levels in the BP and PP were analyzed by using qPCR. The fruits color peels were crushed in liquid nitrogen. Total RNA was isolated using the RNA Trizol kit and cDNA for the qRT-PCR analysis was synthesized from the RNA using PrimeScript RT (Thermo Scientific™). Analyses of qRT-PCR were performed using primer from the nucleotide sequences designed by Primer Premier 5.0 software. The relative transcript levels were calculated compared to the internal control *Alpha Tubulin* using 2^−ΔΔCT^ method for quantitative analysis of the relative expression levels.

### 2.9 Statistical analysis

Data analysis was performed using SPSS software (version 17.0). Data represent the mean ± standard deviation (SD) from at least 3 distinct experiments. Statistical differences were determined using a 2-sample equal variance Student’s *t* test. Statistical significance was defined as *P < 0.05.

## 3 Results

### 3.1 Pigments accumulation in the *Clausena lansium* L. *Skeels* peels

**Figure 1:**
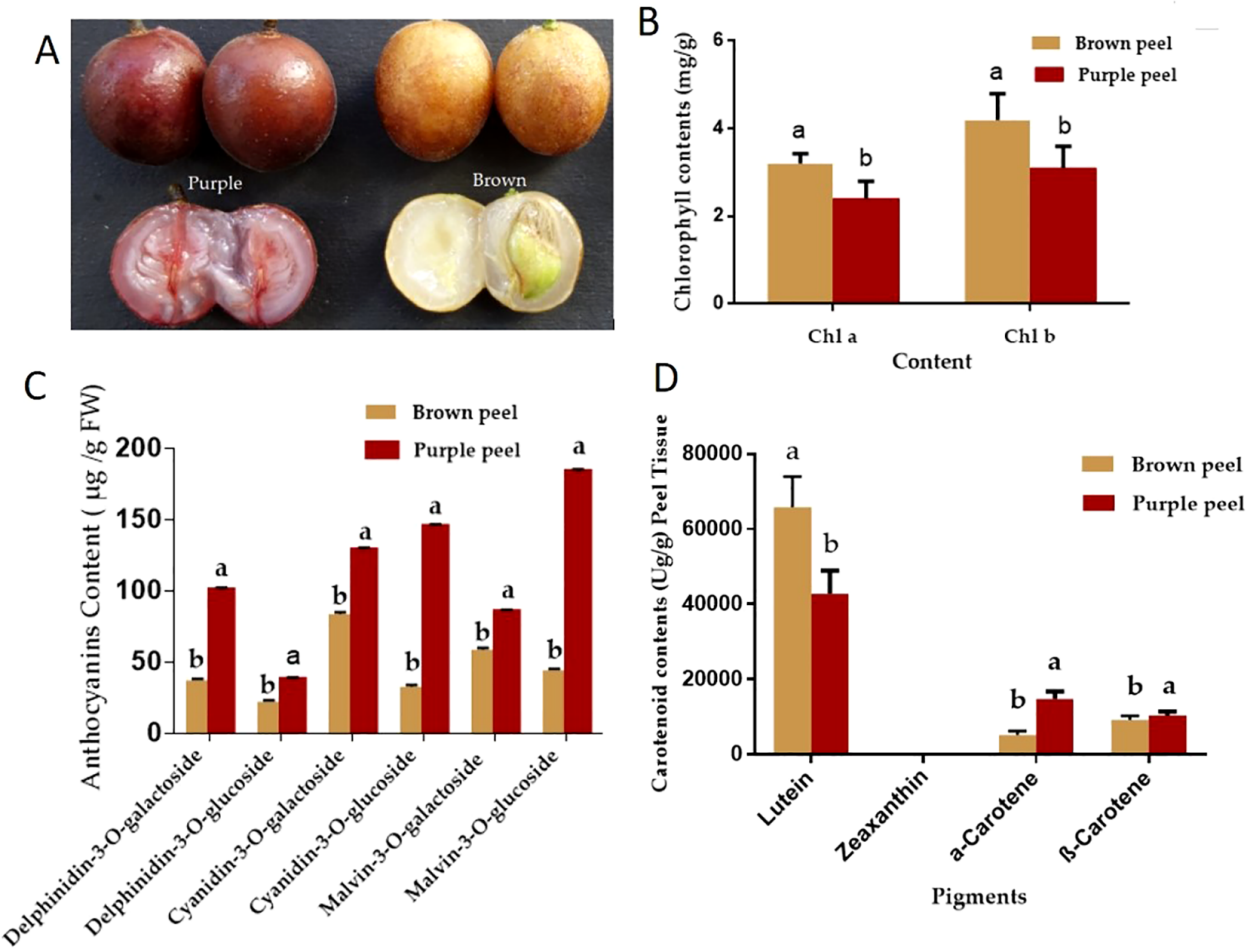
Pigment accumulation analysis of the purple and brown peels. **(A)** cv. *Jixin* fruit brown peel (BP), with cv. *Zijin* purple fruit peels (PP), **(B)**, Chlorophyll contents **(C)** Anthocyanin contents and **(D)** Carotenoid contents. The value with different letters indicates significant differences.

Both BP and PP (**Figure 1A**) contain chlorophyll, carotenoids, and anthocyanins. The levels of chlorophyll a (Chla) and chlorophyll b (Chlb) in the PP are significantly lower than those in the BP (**Figure 1B**). The lower chlorophyll content in PP is beneficial for the peels’ reddish coloration. *Delphinidin-3-O-galactoside*, *delphinidin-3-O-glucoside*, *Malvin-3-O-galactosid*e, *cyanidin-3-O-galactoside*, and *cyanidin-3-O-glucoside* were detected in the two peels (**Figure 1C**). The total anthocyanin content in the PP is significantly higher (about 2.4 times) than in the BP. The contents of the four kinds of Dp in PP were significantly higher than those in BP, especially the *delphinidin-3-O-galactoside* (about 2.7 times) and *mallow-3-O-glucoside* (about 4.2 times). The ratio of Dp/Cy in BP was 1.39, whereas it was 1.49 in PP. It was suggested that PP enhanced the efficiency of anthocyanin biosynthesis and shifted the metabolism balance from the Cy branch to the Dp branch. The deficiency of pelargonidin-based anthocyanin in the two types of peels was intriguing, indicating the loss of Pg branch in anthocyanin biosynthesis pathway (Chen et al., 2022).

The major contents of carotenoids are α-carotene, β-carotene, zeaxanthin, and lutein (Zhou et al., 2007). In the present study, β-carotene, α-carotene, and lutein were detected in the two peels, but zeaxanthin was not detected. The total carotenoids content in BP was about 1.18 times to that of the PP, while the β-carotene and α-carotene in BP were significantly less than that of the PP. The percentage of carotene in total carotenoids increased from 17.9% in BP to 37.1% in PP (**Figure 1D**). Lutein may significantly contribute to the yellow color of BP, while carotene may contribute to the red color of PP.

### 3.2 Identification of the differentially accumulated metabolites

**Figure 2:**
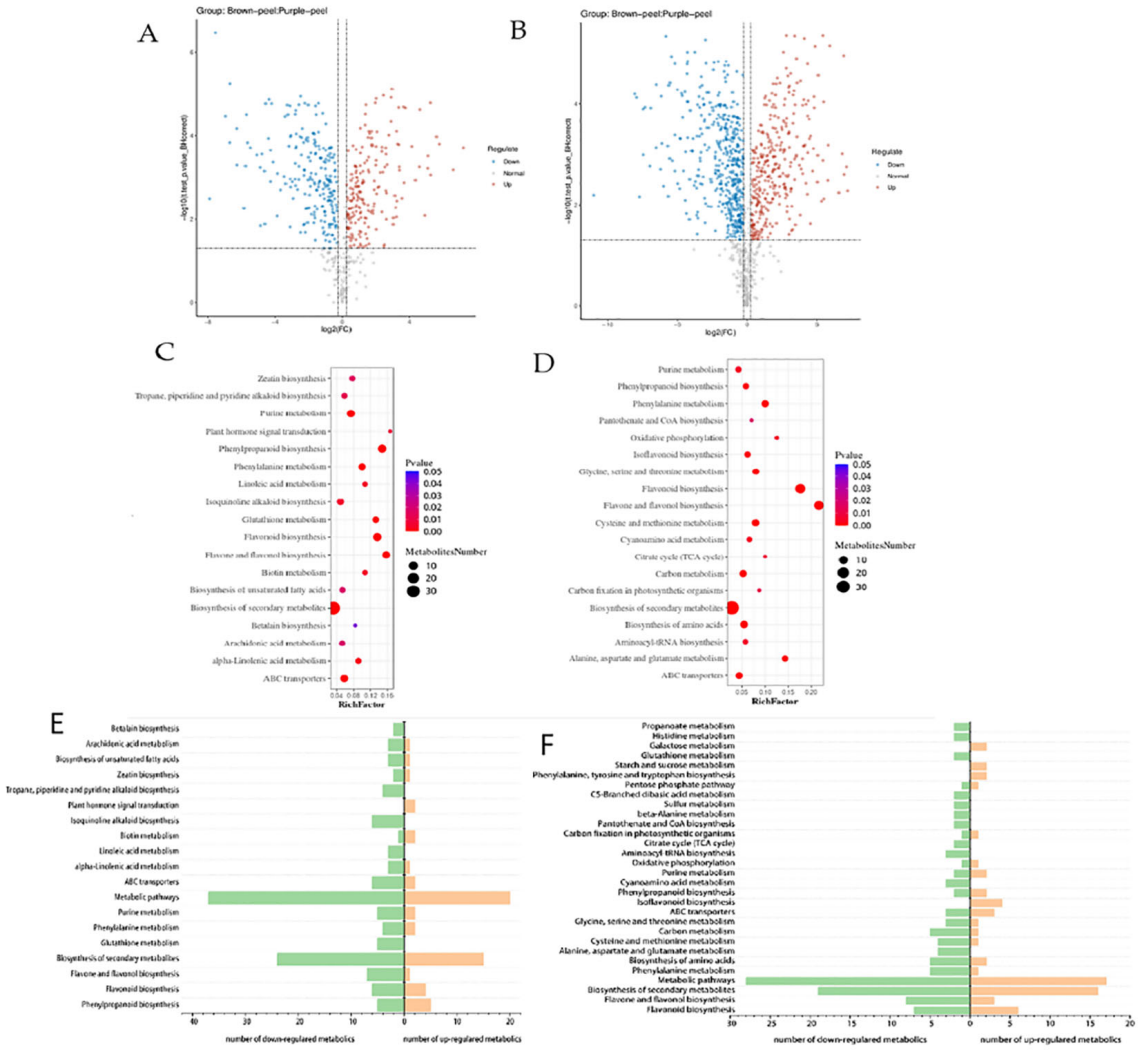
KEGG pathway annotation and volcano plots analysis of the DPMs in BP *vs*. PP. The positive **(A)** and negative **(B)** volcano plots analysis of up and down regulated metabolites, and DPMs enriched KEGG pathways (positive **(C)** and negative **(D)**.Number of up- and down- regulated positive **(E)** and negative **(F)** metabolites enriched in the KEGG pathways. The x-axis represents the richness factor. The color and size of the dots represent p-value and the amount of enriched DPMs, respectively. Rich factor means the ratio of the number of DPMs to the total number of metabolites enriched in a specific category.

The peels were analyzed using HPLC-MS/MS to clarify the changes in secondary metabolites between the two kinds of peels. We identified a total of 373 different metabolites (185 in positive mode and 188 in negative mode) in BP and PP from 49 different pathways. **Figure 2** displays the KEGG pathway annotation and volcano plot analysis of the differentially produced metabolites (DPMs) in PP *vs*. PB. There were 109 positive up-regulated DPMs and 168 positive down-regulated DPMs between the BP and PP samples, as well as 64 negative up-regulated DPMs and 79 negative down-regulated DPMs (**Figure 2A**; **Supplementary Tables 1**, **2**). The KEGG pathways plot (**Figure 2B**) shows that 420 DPMs were filtered, with 277 being positive and 143 being negative. These were chosen based on an absolute log2fold change of ≥1 among the total metabolites with a VIP value of ≥1. **Figures 2C, D**, showed that, biosynthesis of secondary metabolites, flavonoid biosynthesis and phenylpropanoid biosynthesis revealed as the high set DPMs KEGG, Kaempferol; Phloretin; Isosakuranetin; Pinocembrin; Chrysin; Xanthohumol. Moreover, the analysis of the down and up-regulated DPMs was analyzed by KEGG, presenting the positive and negative DPMs (**Figures 2E, F**). Additionally, pathways (19 positive and 30 negative) were annotated to the KEGG database, consisted of “metabolic pathways” (ko01100), “biosynthesis of secondary metabolites” (*ko01110*), “phenylpropanoid biosynthesis” (*ko00940*), “flavonoid biosynthesis” (*ko00941*), “flavone and flavonol biosynthesis” (*ko00944*), etc. These DPMs respectively belonged to “Gluconic acid”, “Citric acid”, “Ferulic acid”, “Abietic acid and Caffeic acid”, Different kinds of flavonoids and flavone and flavonol metabolite were detected in the flavonoid-related pathways (*Ko00941*, *Ko00944*), 23 flavonoids metabolites including 19 kinds of flavone and flavonol showed significantly different production levels between BP and PP samples (**Supplementary Tables 1**, **2**). According to varieties of flavonoids identified, we inferred that three typical biosynthetic branches of flavonoids showed in *Ko00941* do exist in the two peels. Among these are the flavonoids, flavones and flavonols, a category of pigments.

In particular, the 13 flavonoid metabolites with significant differences between PP and BP were as follows: kaempferol, phloretin, isosakuranetin, pinocembrin, chrysin, xanthohumol, epigallocatechin, dihydromyricetin, myricetin, taxifolin, apigenin, quercetin, spermidine, and hesperetin. Differently, these chemicals are connected to pathways that make phenylpropanoid chemicals, flavonoids, and flavone and flavonol chemicals. Comparatively, between PP and BP, there were five up-regulated and eleven down-regulated flavonoid biosynthesis metabolites (**Table 1**). Kaempferol is an important compound as the precursor for flavones, isoflavones, flavonols, and anthocyanins. We found that between the brown and purple peels, the flavonoids metabolites were significantly different (**Table 1**).

**Table 1:** DPMs related to flavonoid biosynthesis between PP and BP.

Metabolites ID	Level	Metabolites	Modes	Pathways	log_2_(Purple/Brown)
**C05903**	Down	Kaempferol	Negative	Phenylpropanoid	-2.428335966
**C05903**	Down	Kaempferol	Positive	Phenylpropanoid	-2.013219107
**C05334**	Down	Isosakuranetin	Negative	Flavonoid/Secondary	0.228413569
**C09827**	Up	Pinocembrin	Negative	Flavonoid	2.143623873
**C10028**	Up	Chrysin	Negative	Flavonoid	2.23297511
**C16417**	Up	Xanthohumol	Negative	Flavonoid	2.520544597
**C12136**	Up	Epigallocatechin	Negative	Flavonoid/Secondary	1.841462132
**C02906**	Down	Dihydromyricetin	Negative	Flavonoid/Secondary	-3.745149235
**C10107**	Down	Myricetin	Negative	Flavonoid/Flavone/Secondary	-0.825690222
**C10107**	Down	Myricetin	Positive	Flavonoid/Flavone/Secondary	-0.603270444
**C01617**	Down	Taxifolin	Negative	Metabolic/Secondary	-4.759337863
**C01477**	Up	Apigenin	Negative	Flavonoid/Flavone/Secondary/Metabolic	1.245204568
**C00389**	Down	Quercetin	Negative	Flavonoid/Flavone/Secondary/Metabolic	-0.473539196
**C00389**	Down	Quercetin	Positive	Flavonoid/Flavone/Secondary/ Metabolic	-0.866539077
**C01709**	Down	Hesperetin	Negative	Flavonoid	-0.475934812
**C01709**	Down	Hesperetin	Positive	Flavonoid	-1.277666085

*All the numbers in bold indicate the flavonoid differentially Produced Metabolites.

### 3.3 Differentially expressed genes of *Clausena lansium* L. *Skeels* peels

**Figure 3:**
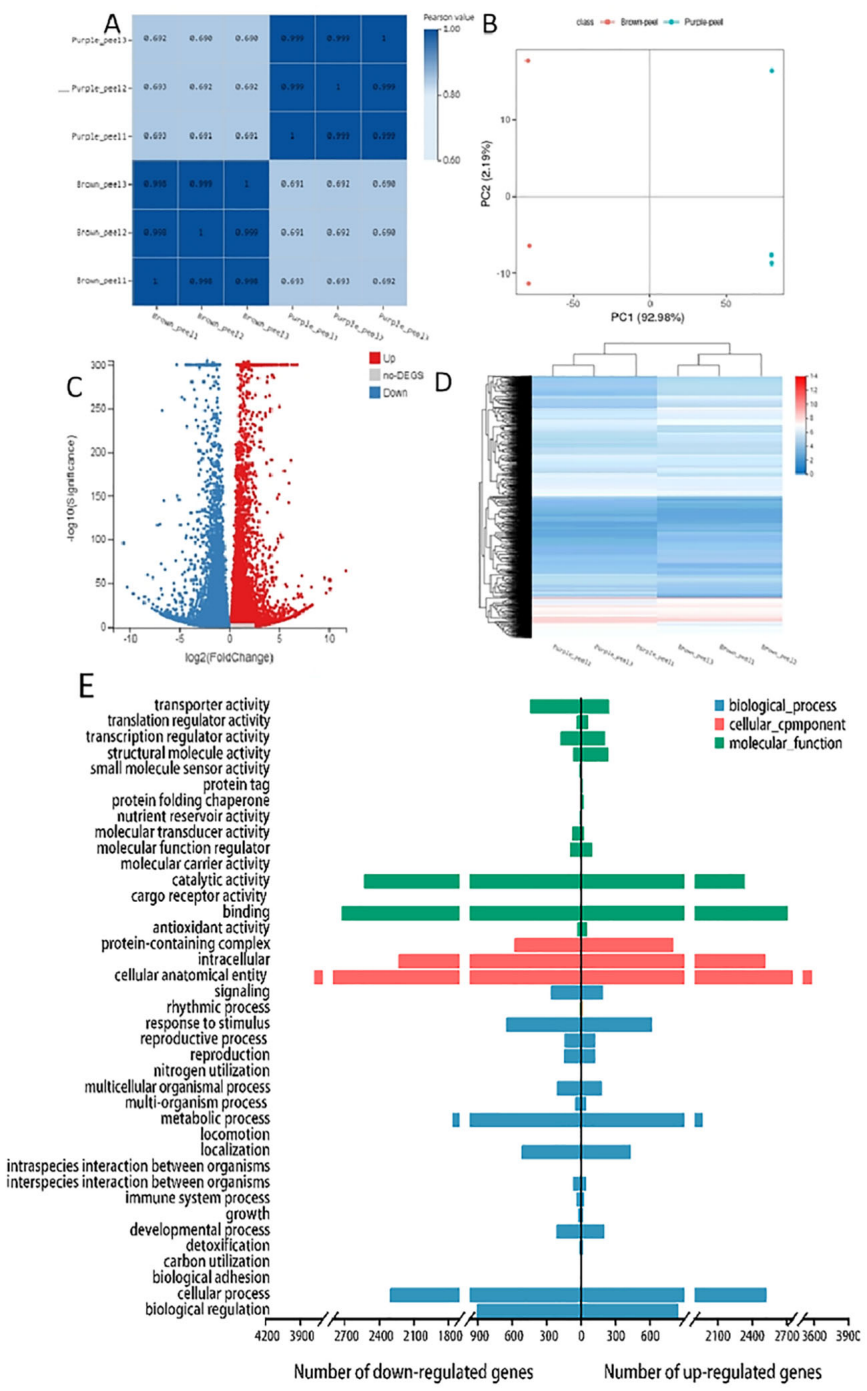
RNA-Seq gene expression distribution and transcriptome profiling of BP and PP. **(A)**, The value of the relationship between the sample correlation coefficient. **(B)** Heat map clustering of DEGs showed the accumulation in peels samples, **(C)** The principal component analysis (PCA), **(D)** The volcano plots accumulation of metabolites of up-regulated and down-regulated DEGs, **(E)** GO bar plot indicating the number of up- and down- regulated DEGs.

According to the reference transcriptome, the number of expressed genes detected was 23,920, of which the known genes were 19,915 and 4,005 new genes were predicted; a total of 25,430 new transcripts were detected, of which 21,379 belonged to new alternative splicing isoforms of known protein-coding genes, and 4,051 belonged to transcripts of new protein-coding genes. Differentially expressed genes (DEGs) were selected by an absolute log_2_fold-change ≥1 and compared to two databases (KEGG, and GO) to determine their potential functions. Between the samples the value of the relationship between the sample coefficients correlated that means correlation coefficients has similar strength and direction of the linear relationships between pairs of peels (**Figure 3A**). In addition, a heat map clustering of DEGs showed the accumulation pattern of the samples (**Figure 3B**), indicating that the genes in the same cluster may have similar biological functions. A larger value of PC negative meant a higher degree of genetic variation among different varieties. The PCA results presented significant differences between the two peels showed the value of Q2Y was 1, indicating the reliability of the data (**Figure 3C**). In total, 12990 DEGs were selected, and volcano revealed that there were 6570 up-regulated and 6420 down-regulated DEGs between the BP and PP samples (**Figure 3D**). Moreover, among the number of up-regulated and down-regulated DEGs of biological processes, approximately 2300 genes are down-regulated and more than 2500 genes are up-regulated in cellular processes. Approximately more than 3700 genes are down-regulated and 3400 are up-regulated in cellular anatomical entities. In the molecular function, approximately 2700 genes are down-regulated and 2500 are up-regulated as molecular function (**Figure 3E**). The fragments per kilobase of transcript per million fragments (FPKM) gene expression patterns identify the function of DEGs in the formation of fruit peel coloration, three categories were classified, including molecular function (MF), cellular component (CC), and biological process (BP), according to GO classifications.

The most DEGs enriched GO terms between PP and BP were the intracellular, catalytic activity, binding, cellular anatomical entity, cellular process, metabolic process, and other functional categories (**Figure 3E**). A total of 10,866 DEGs in 137 KEGG pathways were enriched, which including “Carbon metabolism” (*ko01200*), “Phenylpropanoid biosynthesis” (*ko00940*), Flavonoid biosynthesis’’ (*ko00941*) “Anthocyanin biosynthesis” (*ko00942*), “Glycolysis/Gluconeogenesis “(*ko00010*) and so on (**Supplementary Table 5**). The log_2_ (Purple_peel/Brown_peel) of flavonoid biosynthesis” *(ko00941*), and anthocyanin biosynthesis (*ko00942*) between the BP *vs* PP showed up-regulated. Such as *anthocyanidin 3-O-glucosyltransferase 7* (Maker00016682), *Flavonol 3-O-glucosyltransferase* (Maker00016633), *DMR6-like oxygenase* (Maker00017279), *Cinnamate 4-hydroxylase* 1 (Maker00017732), *Stemmadenine-O-acetyl- transferase* (Maker00019099), *2-oxoglutarate 3-dioxygenase* (Maker00020222) and *phenylalanine ammonia-lyase* (Maker00017942) etc.

**Figure 4:**
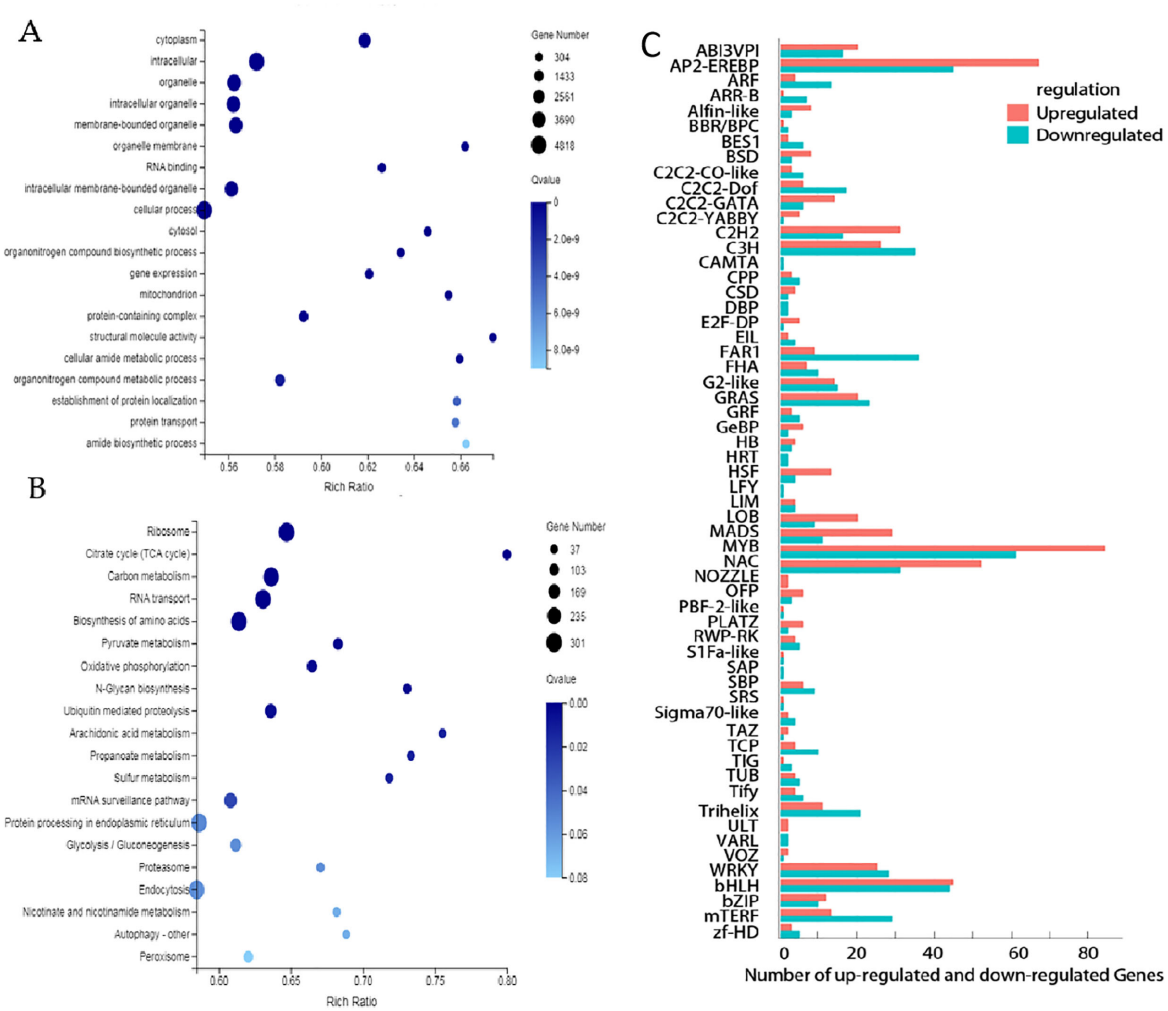
Differentially expressed genes between BP and PP. **(A)** The GO enrichment analysis, **(B)** the KEGG enrichment analysis, **(C)** Numbers of up- and down- regulated transcription factors (TFs).

The GO bar plot indicated that the number of DEGs significantly involved in metabolic process, cellular processes, cells, cell parts, catalytic action, RNA binding, etc. (**Figure 4A**). KEGG analysis revealed that Ribosome (*ko03010*), Carbon metabolism (ko01200), RNA transport (*ko03013*) and Biosynthesis of amino acids (*ko01230*) were the top four significantly changed pathways (**Figure 4B**). In addition, transcription factors (TFs) differently expressed between BP and PP were identified. 170 genes belong to Myeloblastosis (MYB), AP2-(Ethylene-responsive element binding proteins) (133), Basic helix-loop-helix (bHLH) (112), N-acetylcysteine (NAC) (107), C3H (63), C2H2 (49), Basic-leucine zipper (bZIP) (23) and other families (**Figure 4C**). The higher number of AP2, MYB, and bZIP transcription factors significantly up-regulated and down-regulated (**Figure 4B**). All these factors play an important role in regulation and promoting secondary metabolism in anthocyanins and flavonoids.

### 3.4 Correlation analysis of the relationship between genes and metabolites involved in flavonoid biosynthesis of *Clausena lansium* L. *Skeels*

Among these genes, several flavonoid related genes were identified and showed correlations with biosynthesis of flavonols, flavones and anthocyanins. Based on metabolome and transcriptome studies with the KEGG database, a pathway map encompassing metabolites and structural genes linked to flavonoid and anthocyanin production were identified in this study. The expression levels of *Flavanone 3-hydroxylase* (*F3H*) (Maker00020222, Maker00022711, and Maker00007403), *Flavonol synthase* (*FLS*) (Maker00012832), *Chalcone synthase* (*CHS*) (Maker00005905), and *flavonoid-3-O-glucosyltransferase* (*UFGT*) (Maker00008406) showed significantly different between two peels. The expression of *CHS*, and *F3H* increased higher in the PP than in BP. These gene was found to correlate with naringenin, and kaempferol showing the existence of flavanone glycoside that displays strong anti-inflammatory and antioxidant activities (Zhaoa et al., 2004; Chang et al., 2018) (**Supplementary Table 3**). Moreover, either the BP or PP are probably masked by different proportion of flavone, flavonoid, anthocyanins and phenylpropanoid metabolites. Such as kaempferol, naringenin, pinocembrin, chrysin, xanthohumol, epigallocatechin, Dihydromyricetin, myricetin, taxifolin, apigenin, and quercetin. Among the identified genes, more than 9 of flavonoid related genes were identified and showed correlations with these identified flavonoid and anthocyanins metabolites. Thus the *F3H*, *DFR*, *UFGT* and *FLS* were positively correlated with the content of naringenin, kaempferol, dihydromyricetin, apigenin, luteolin, tricetin and quercetin, indicating these genes are important for the flavonoid biosynthesis and coloration of BP and PP.

### 3.5 The branching characters of anthocyanin biosynthesis pathway in the *Clausena lansium* fruits peels

Anthocyanin biosynthesis pathway (ABP) have 3 major branches: cyanidin (Cy), pelargonidin (Pg), and delphinidin (Dp) (Cho et al., 2020). The total anthocyanin content in PP is about 2.4 times to that of BP. The Dp/Cy ratio was 1.39 and 1.49 in BP and PP, respectively. However, in PP *F3′H* and *F3′5′H* highly expressed, which catalyze a larger portion of the DHK to the Dp branch, while more DHK move downstream towards the Cy branch in BP. Our findings demonstrate the intricacy of altering a biosynthetic pathway with many branch points to distinct end products and the complicated interactions between the kind and concentration of flavonol co-pigments and anthocyanin pigments in the fruit color. The crucial enzyme competition between F3’H and F3’5’H determining the Dp/Cy ratio, has far-reaching consequences that affected not only anthocyanins but also other flavonoid pathway branches.

Furthermore, the CHS gene exhibited increased expression in PP, potentially leading to a higher concentration of naringenin chalcone (C00509), which is responsible for anthocyanin accumulation in PP. There was competition up and downstream of Cy, Dp, DHM, and DHK for Cy and Pg branches (Johnson et al., 1999; Li et al., 2017; Haselmair-Gosch et al., 2018). This competition indicated that the metabolic flux of ABP is controlled by substrate competition between the FLSs and the DFRs. The competition between *FLS and DFR* resulted in the production of flavonol aglycones, such as kaempferol, myricetin, and quercetin (Kapoor et al., 2022), which could potentially alter the color differences between cv. *Jixin* and cv. *Zijin*. The competition between *FLS* and *DFR* for dihydroflavonols that creates branching of critical branch point separating the flavonol and anthocyanin biosynthetic pathways. Previous studies showed that, *DFR* role can cause Cy formation causing the red coloration in the spathes of *Philodendron* (Strack and Wray, 2017). In different *Senecio cruentus* cultivars, there were substrates competition between FLS and DFR caused blue pigmentation elimination among the cultivars (Jin et al., 2016), which seems to be the likely reason for the color change in the BP.

Moreover, the accumulation of Cy and Dp and the lack of Pg in the peels indicated that the DFR of *Clausena lansium L. Skeels* can catalyze DHM and DHQ efficiently and cannot utilize DHK as substrate, the Pg biosynthesis branch was blocked. The *F3′H*, *F3′5′H* and *DFR* were expressed higher in PP. From this results, we believed that the higher expression of *F3′H* and *F3′5′H* coupled with the higher expression of *DFRs* can catalyze DHK conversion to DHQ and DHM more efficiently and enhance the biosynthesis of leucocyanidin and leucodelphinidin. The significant decrease of DHM content in PP also suggested the higher efficiency conversion of DHM to leucodelphinidin, which leading to the higher accumulation of Dp. Moreover, the PP has higher expression of *Quercetin 3-O-glucosyltransferase* (UFGT) which is the prerequisite for acylation responsible for stabilization and the imparting of a purple color to the anthocyanin (Fukuchi-Mizutani et al., 2003).

**Figure 5:**
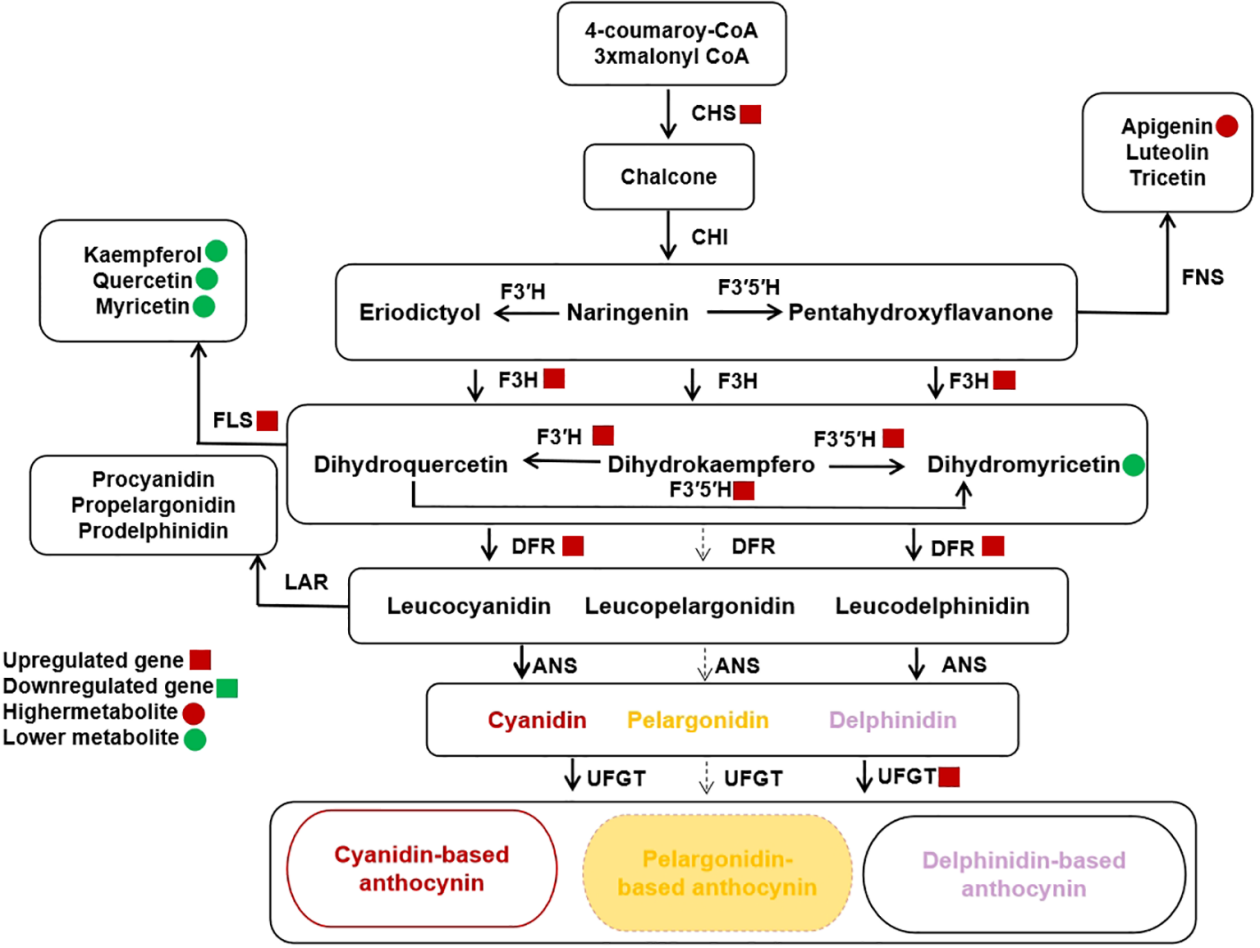
Schematic of Anthocyanin Biosynthetic Pathway (ABP) branches relevant to *Clausena lansium* (Lour.) *Skeels* Fruit Peels Color. Anthocyanidins (cyanidin, and delphinidin) are modified from leucoanthocyanidins. Brown peels gentians contain gentiocyanin, and purple gentians mainly Leucodelphinidin, whereas both accumulates exclusively pelargonidin. Abbreviations: CHI, chalcone isomerase; CHS, chalcone synthase; *FLS* flavonol synthase, DFR, dihydroflavonol 4-reductase; DHK, Dihydrokaempferol; DHM, Dihydromyricetin; DHQ, Dihydroquercetin; F3’5’H, flavonoid 3’,5’-hydroxlase and anthocyanidin synthase (ANS). The small red circles and squares indicate metabolites and log2 of the up-regulated genes of the peels respectively.

However, pelargonidin-based anthocyanin is not detected in both brown and purple fruits peels (**Figures 1C**, **5**). This maybe because its *DFR* does not catalyze DHK. In some species the DFRs may not catalyze DHK or only has low chemical affinity with DHK (Miosic et al., 2014). Such as the DFR of *chrysanthemum* can catalyze DHM, but cannot utilize DHK as a substrate (Seo et al., 2007; He et al., 2013). In addition, the Pg loss may include the loss-of-function mutations of any pathway, including any of the three proteins (MYBs, WRKYs and bHLHs) that regulate the expression of the enzyme-coding genes, as well as cis-regulatory mutations that down-regulate any of the pathway enzymes (Li, 2014; Dalman et al., 2017; Li et al., 2020). Different from our findings, *Prunus mume* accumulated pelargonidin to produce high *DFR* expression but *F3’H*, and *FLS* genes expression down regulated (Qiu et al., 2022). Moreover, the overexpression of *FLS* in tobacco flowers has resulted in increased flavonol content and decreased anthocyanin content (Park et al., 2020).

### 3.6 qPCR validation

**Figure 6:**
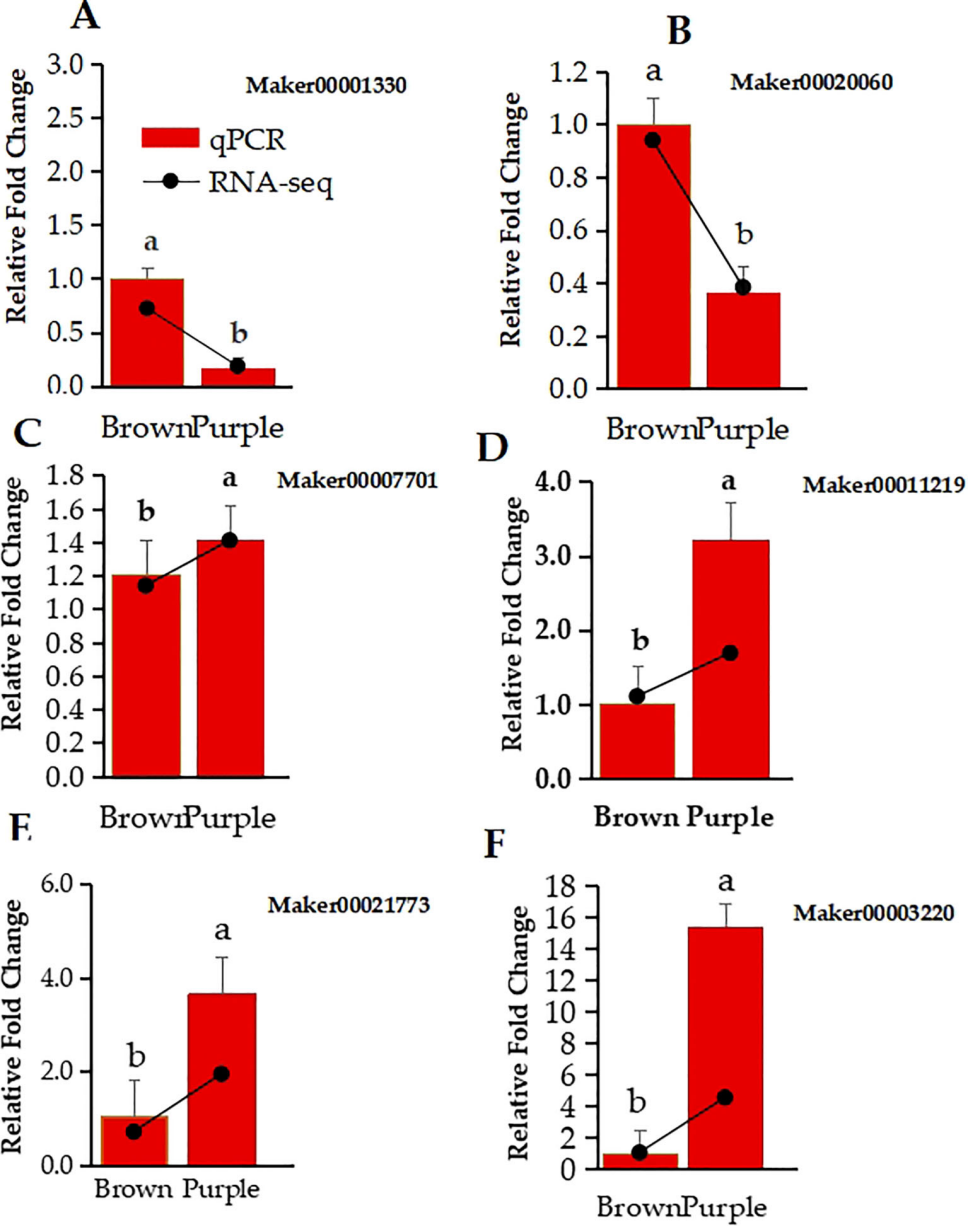
Relative expression levels of 6 DEGs in *Clausena lansium* L. *Skeels* between brown and purple Peels were determined by qRT-PCR and RNA-seq. **(A–F)** show the qPCR relative fold change in bar graph and log2fold change in line of RNA-Seq data expression of FYVE domains-containing protein 1, Expansin-A4, DELLA protein GAI, transcription factor MYB3R-1, probable auxin efflux carrier component 1c, and polygalacturonase. The lower case a and b indicate the significant difference between the two expression values.

To validate the RNA-Seq results, six related genes consisting of *FYVE domains-containing protein 1* (Maker00001330), *Expansin-A4* (Maker00021773), *DELLA protein GAI* (Maker00011219), MYB *Transcription factor MYB3R-1* (Maker00003220), *Probable auxin efflux carrier component 1c* (Maker00020060), and *Polygalacturonase* (Maker00007701) were selected by their roles in flavonoids and anthocyanins biosynthesis pathways and their high expression values, and their relative expression levels in the BP and PP phenotypes were analyzed by using qPCR. The qPCR compared with the transcriptome result of the 6 selected expressed genes confirmed the confidence of the RNA-seq data. More importantly, the qPCR relative fold change expression of *FYVE domains-containing protein 1* (**Figure 6A**), *Expansin-A4* (**Figure 6B**), *DELLA protein GAI* (**Figure 6C**), *Transcription factor MYB3R-1* (**Figure 6D**) *probable auxin efflux carrier component 1c* (**Figure 6E**), and *Polygalacturonase* (**Figure 6F**) was similar to log2fold change of RNA-Seq data. (**Figure 6**).

## 4 Discussion

The cyanidin (Cy), pelargonidin (Pg), and delphinidin (Dp) pathways are the three major branching of anthocyanin biosynthesis pathway that are responsible for different colorful fruits, flowers and leaves (Zhang et al., 2021). Studies have revealed that different species have evolved to develop multiple and different regulatory mechanisms that form the anthocyanin branching pathways (Zeng et al., 2024; Prasad et al., 2010; Luo et al., 2022). This study analyzed pigments accumulation and the regulatory mechanism of anthocyanin branching pathway by integrating metabolomics and transcriptomic of *Clausena lansium* L. *Skeels* fruit peels based on complex accumulation content, biosynthesis, distribution and types of intracellular pigments. Similarly in Persimmon, the differences of anthocyanin, carotenoid and chlorophyll content were the main reasons for the difference between the two fruit peel color (Ye et al., 2022). Likewise, in ornamental kale, the bicolor formation was revealed to be caused by the anthocyanin and chlorophyll contents (Ren et al., 2019).

In general, the fruit peels color difference was frequently related to the composition and concentration of anthocyanidins and carotenoids. Our study showed that the brown peel carotenoid biosynthesis content was higher in the lutein and purple peel was higher in β-carotene, and α-carotene (**Figure 1**). However, both fruit peels accumulate no zeaxanthin, which may be the reason that both peels have lipid-free zeaxanthin for ROS released from the Chl binding complexes (Ye et al., 2022). The lack of zeaxanthin had a much stronger impact on chlorophyll, thus decreasing photoprotection in PP. Different from our studies, abundance levels of violaxanthin, antheraxanthin, and zeaxanthin generated yellow color in the majority of fruits (Dall'Osto et al., 2010; Roca and Pérez-Gálvez, 2021). These color changes were probably caused by dynamic changes in carotenoid accumulation. In addition, the BP to PP transformation process of *Skeels* fruits peels may be formed by the combination of carotenoids along with chlorophylls. In addition we found the most common anthocyanidin compounds included pelargonidin, cyanidin, and delphinidin. Previous research had shown that these anthocyanidins regulated apples and peaches pigments biosynthesis (Johnson et al., 1999; Li et al., 2017; Haselmair-Gosch et al., 2018). Thus the differences in the peel color were formed with anthocyanin degrading in the brown and the levels of chlorophyll in the PP were significantly lower than those in the BP (**Figures 1B, C**). The cyaniding along with delphinidin induced variation in flower shades (Li et al., 2010; Qiu et al., 2013). Higher abundance levels of three metabolites including cyanidin-3,5-o-diglucoside, delphinidin-3-o-galactoside, and pelargonidin-3,5-o-diglucoside may be more beneficial to facilitate different fruit coloration in *Clausena lansium* L. *Skeels*. The reason was that color intensity and stability induced by anthocyanidin compounds altered BP to PP reddish color and other factors such as total anthocyanin content in PP more than that of BP. Utilizing the up and downstream of Cy, Dp, DHM, and DHK for Cy and Pg branches (Johnson et al., 1999). Specifically, cyanidin, delphinidin, and pelargonidin were found to be unstable in BP (Kapoor et al., 2022).

In the anthocyanins pathway, not all DFRs type catalyze all the three dihydroflavonols as substrates (Liu et al., 2017). Such as DFRs from *Ipomoea batatas dihydroflavonol-4-reductase* (IbDFR) catalyzes only the DHK but not DHQ and DHM (Johnson et al., 1999; Li et al., 2017; Haselmair-Gosch et al., 2018). Buckwheat (*Fagopyrum esculentum*) *dihydroflavonol-4-reductase 2* (FeDFR2) utilizes both the DHQ and DHM but not the DHK as a substrate as they are classified into the Asn-type DFR, due to their 134th amino acid residues (Katsu et al., 2017; Liu et al., 2017; Lim et al., 2020). Consistent in our findings, the metabolism balance between Dp branch and Cy branch was un-broken in the two peels, while the metabolism intensity was enhanced in purple peels. This is the reason that the total anthocyanin content in PP is about two times to that of BP. However, pelargonidin-based anthocyanin was not detected in brown and purple fruit peels (**Figures 1C**, **5**). This maybe because it’s DFR does not catalyze DHK. The DFR of *Clausena lansium* L. *Skeels* may be Asp-type DFR which are highly specific for DHQ and DHM rather than tAsn-type DFR which catalyze the conversion of all three dihydroflavonols (DHK, DHQ, and DHM) (Johnson et al., 1999; Li et al., 2017; Haselmair-Gosch et al., 2018).

Our studies showed that, the three colorless dihydroflavonols are substrates of flavonol biosynthesis that are catalyzed by *FLS* (**Figure 5**). This may be the competition between *FLS* and *DFR* can modify the metabolic substrate and alter peel colors as revealed in *Chrysanthemum* (Kapoor et al., 2022). The presence of *FLS* in both peels to produce flavonol aglycones such as kaempferol and quercetin (**Supplementary Table 3**). However, the FLS up regulated in PP than BP is separated from the anthocyanins for more flavonol, thus in the flavonoid biosynthesis pathway, the production level of chrysin, Apigenin, pinocembrin, and xanthohumol increased in purple peels significantly. This lead to competition between *FLS* and *DFR* for dihydroflavonols that creates critical branch point separating the flavonol and anthocyanin biosynthetic pathways (Mou et al., 2015; Park et al., 2019). The changes of Dp/Cy ratio, which maybe caused by *F3’5’H*, and the changes of total anthocyanin maybe caused by the increase of the gene expression including *F3’5’H*, and *UFGT*. The biosynthesis of proanthocyanidin substances is the structural genes including CHS, and F3H. In our studies we find out that the expression of *CHS*, *F3H* were upregulated in the PP than in BP. At lateral *Clausena Lansium* L. *Skeels* fruits color development stages genes up regulation might be in response to a higher abundance of naringenin, Apigenin, and Dihydrokaempferol, and the biosynthetic genes *F3′H* and *F3′5′H.* Hence, fruit could display reddish color. This to say that, the conversion of naringenin played a significant role as apigenin level increased (**Table 1**) to form *CHS* enzyme tilting anthocyanins branches may also influence PP color changes (Li et al., 2010; Qiu et al., 2013).

Studies have shown that, the accumulation of cyanidin-based and delphinidin-based anthocyanins form moderately change in visual colors of anthocyanins, and the biosynthetic genes *F3′H* and *F3′5′H* increase of anthocyanin accumulation (Zhang et al., 2021). The *F3′H* belongs to the *CYP75B* subfamily that converts dihydrokaempferol to dihydroquercetin which is the precursor of cyanidin-based anthocyanin (Castellarin et al., 2006). They are characterize key necessary for accumulation of anthocyanin in fruit, and differential regulation of *F3′H* and *F3′5′H* that contributes to the ratio of cyanidin and delphinidin in different fruit species. Similarly to our findings the ratio of cyanidin to delphinidin was about 3/2 and a higher amount of delphinidin was accumulated in PP. Our findings shows that the relative expression of *F3’H* and *F3′5′H* function in the color differences between PP and BP. In *Actinidia arguta* var. *purpurea* (Red Kiwi) the expression of *F3′H2* was very low at the color-change stage but was complemented by the high expression of *F3′5′H* which may explain the similar level of cyanidin-based and delphinidin-based anthocyanin (Peng et al., 2019).

Actually, the most enriched DPMs and DEGs distributed in many other pathways, such as biosynthesis of amino acids, signal transduction, metabolism of glucose and so on, which have correlation with physiological processes. The relationship between these anthocyanins and flavonoid biosynthesis in *Clausena lansium* L. *Skeels* is useful for plant breeders to seek to generate novel fruit colors. The anthocyanin branching point genes, including *F3′5′H*, *F3’H, FLS* and *DFRs*, are all crucial gene resources for the future transgenic breeding of *Clausena lansium* L. *Skeels*.

## 5 Conclusion

The current study discovered that different types of *Clausena lansium* L. *Skeels* peels exhibit distinct patterns of metabolite activity accumulation, which reveal the process of color formation. Carotenoid and anthocyanin pigments play a key role in determining the color of *Clausena lansium* L. *Skeels* peels. They possess antioxidant properties and potential protection against diseases, making their biosynthesis essentially important. Purple peels had a higher total chlorophyll and anthocyanin content than brown peels. The combined analysis of metabolites and metabolomes revealed that purple peels have more α-carotene and β-carotene but less Chla, Chlb, and lutein than brown peels. This led to the incapability of catalyzing chalcone into naringenin, which ultimately resulted in a pale yellow peel color. The variation in coloring process among the two peels with different colors originated from the DHQ branching point. The increased flow toward the Cy branch resulted the two DFRs used DHQ as their substrate, causing the accumulation of Cy in the purple peel. The competition between *F3’H*, and *F3’5’H*, may determine the ratio of Dp/Cy, and the substrate specificity of *DFR* result in a lack of pelargonidin in the peels. The high levels of *F3’H*, *F3’5’H*, and *UFGT* in the cv. Zijin peel, as well as the use of DHQ by the two DFRs as their substrate, possibly lead to the accumulation of Cy in the purple peels. Understanding these biosynthetic pathways allows for biotechnological applications for the nutritional profile of fruits through metabolic engineering or breeding strategies aimed at increasing antioxidant content.

## Data Availability

The data presented in the study are deposited in the NCBI repository, submission number SUB15012360.
