# Supplementary material for: Metabolome and transcriptome analysis reveal the pigments biosynthesis pathways in different color fruit peels of Clausena lansium L. Skeels
Source: Front Plant Sci. 2025 Jan 31;15:1496504. doi: 10.3389/fpls.2024.1496504 (PMC11825772; doi:10.3389/fpls.2024.1496504)
Supplement: Supplementary file 1 [file Table1.docx]

Metabolome and Transcriptome Analysis Reveal the Pigments Biosynthesis Pathways in Different Color Fruit Peels of *Clausena Lansium* L. *Skeels*

Zhao Zhichang ^1^, Mark Owusu Adjei ^2^ , Luo Ruixiong ^1^, Yu Huaping ^1^, Pang Yali ^1^, Wang Jian^1^, Zhang Yu ^3^*, Ma Jun ^2^*, Gao Aiping ^1^*

**Corresponding authors emails:**

**Zhang Yu**: [gxrzszy0624@163.com](mailto:gxrzszy0624@163.com)

*Guangxi Subtropical Crops Research Institute, Nanning, Guangxi, 530001.*

**Ma Jun**: [41185@sicau.edu.cn](mailto:41185@sicau.edu.cn)

*Landscape Architecture College of Sichuan Agricultural University, Chengdu, Sichuan, 611130.*

**Gao Aiping**: [aipinggao@catas.cn](mailto:aipinggao@catas.cn)

*Tropical Crops Genetic Resources Institute Chinese Academy of Tropical Agricultural Sciences, Haikou, Hainan, 571101.*

**Authors**

**Zhao Zhichang** - *Tropical Crops Genetic Resources Institute Chinese Academy of Tropical Agricultural Sciences, Haikou, Hainan, 571101.*

**Mark Owusu Adjei** - *Landscape Architecture College of Sichuan Agricultural University, Chengdu, Sichuan, 611130.*

**Luo Ruixiong -** *Tropical Crops Genetic Resources Institute Chinese Academy of Tropical Agricultural Sciences, Haikou, Hainan, 571101.*

**Yu Huaping** - *Tropical Crops Genetic Resources Institute Chinese Academy of Tropical Agricultural Sciences, Haikou, Hainan, 571101.*

**Pang Yali -** *Tropical Crops Genetic Resources Institute Chinese Academy of Tropical Agricultural Sciences, Haikou, Hainan, 571101.*

**Wang Jian -** *Tropical Crops Genetic Resources Institute Chinese Academy of Tropical Agricultural Sciences, Haikou, Hainan, 571101.*

**Content**

**Supplementary Table 1**: Positive metabolites in relation to fruit peel coloration.

**Supplementary Table 2**: Negative metabolites in relation to fruit peel coloration.

**Supplementary Table 3**. Negative regulation level of metabolites between BP and PP.

**Supplementary Table 4**. Positive regulation level of metabolites between BP and PP.

**Supplementary Table 5:** The correlation relationship between genes and metabolites involved in flavonoid and anthocyanin biosynthesis Peels.

**Supplementary Table 1**: Positive metabolites in relation to fruit peel coloration

| No. | Pathway | Count | All metabolites with pathway annotation (Count. All) | P value | Pathway.ID |
| --- | --- | --- | --- | --- | --- |
| 1 | [Phenylpropanoid biosynthesis](D:/Prof%20Gao/%E9%BB%84%E7%9A%AE%E6%95%B0%E6%8D%AE%E6%96%87%E7%AB%A0/pos_Brown-peel_Purple-peel-quant-identification/enrich.htm#gene1) | 10 | 68 | 2.671106e-08 | map00940 |
| 2 | [Flavonoid biosynthesis](D:/Prof%20Gao/%E9%BB%84%E7%9A%AE%E6%95%B0%E6%8D%AE%E6%96%87%E7%AB%A0/pos_Brown-peel_Purple-peel-quant-identification/enrich.htm#gene2) | 10 | 74 | 6.144057e-08 | map00941 |
| 3 | [Flavone and flavonol biosynthesis](D:/Prof%20Gao/%E9%BB%84%E7%9A%AE%E6%95%B0%E6%8D%AE%E6%96%87%E7%AB%A0/pos_Brown-peel_Purple-peel-quant-identification/enrich.htm#gene3) | 8 | 51 | 4.067324e-07 | map00944 |
| 4 | [Biosynthesis of secondary metabolites](D:/Prof%20Gao/%E9%BB%84%E7%9A%AE%E6%95%B0%E6%8D%AE%E6%96%87%E7%AB%A0/pos_Brown-peel_Purple-peel-quant-identification/enrich.htm#gene4) | 39 | 1237 | 7.785697e-07 | map01110 |
| 5 | [Glutathione metabolism](D:/Prof%20Gao/%E9%BB%84%E7%9A%AE%E6%95%B0%E6%8D%AE%E6%96%87%E7%AB%A0/pos_Brown-peel_Purple-peel-quant-identification/enrich.htm#gene5) | 5 | 38 | 0.0001565259 | map00480 |
| 6 | [Phenylalanine metabolism](D:/Prof%20Gao/%E9%BB%84%E7%9A%AE%E6%95%B0%E6%8D%AE%E6%96%87%E7%AB%A0/pos_Brown-peel_Purple-peel-quant-identification/enrich.htm#gene6) | 6 | 60 | 0.000162649 | map00360 |
| 7 | [Purine metabolism](D:/Prof%20Gao/%E9%BB%84%E7%9A%AE%E6%95%B0%E6%8D%AE%E6%96%87%E7%AB%A0/pos_Brown-peel_Purple-peel-quant-identification/enrich.htm#gene7) | 7 | 95 | 0.0003189694 | map00230 |
| 8 | [Metabolic pathways](D:/Prof%20Gao/%E9%BB%84%E7%9A%AE%E6%95%B0%E6%8D%AE%E6%96%87%E7%AB%A0/pos_Brown-peel_Purple-peel-quant-identification/enrich.htm#gene8) | 57 | 2701 | 0.0004071454 | map01100 |
| 9 | [ABC transporters](D:/Prof%20Gao/%E9%BB%84%E7%9A%AE%E6%95%B0%E6%8D%AE%E6%96%87%E7%AB%A0/pos_Brown-peel_Purple-peel-quant-identification/enrich.htm#gene9) | 8 | 137 | 0.0005846436 | map02010 |
| 10 | [alpha-Linolenic acid metabolism](D:/Prof%20Gao/%E9%BB%84%E7%9A%AE%E6%95%B0%E6%8D%AE%E6%96%87%E7%AB%A0/pos_Brown-peel_Purple-peel-quant-identification/enrich.htm#gene10) | 4 | 44 | 0.002973309 | map00592 |
| 11 | [Linoleic acid metabolism](D:/Prof%20Gao/%E9%BB%84%E7%9A%AE%E6%95%B0%E6%8D%AE%E6%96%87%E7%AB%A0/pos_Brown-peel_Purple-peel-quant-identification/enrich.htm#gene11) | 3 | 28 | 0.006357708 | map00591 |
| 12 | [Biotin metabolism](D:/Prof%20Gao/%E9%BB%84%E7%9A%AE%E6%95%B0%E6%8D%AE%E6%96%87%E7%AB%A0/pos_Brown-peel_Purple-peel-quant-identification/enrich.htm#gene12) | 3 | 28 | 0.006357708 | map00780 |
| 13 | [Isoquinoline alkaloid biosynthesis](D:/Prof%20Gao/%E9%BB%84%E7%9A%AE%E6%95%B0%E6%8D%AE%E6%96%87%E7%AB%A0/pos_Brown-peel_Purple-peel-quant-identification/enrich.htm#gene13) | 6 | 122 | 0.006536918 | map00950 |
| 14 | [Plant hormone signal transduction](D:/Prof%20Gao/%E9%BB%84%E7%9A%AE%E6%95%B0%E6%8D%AE%E6%96%87%E7%AB%A0/pos_Brown-peel_Purple-peel-quant-identification/enrich.htm#gene14) | 2 | 12 | 0.01114212 | map04075 |
| 15 | [Tropane, piperidine and pyridine alkaloid biosynthesis](D:/Prof%20Gao/%E9%BB%84%E7%9A%AE%E6%95%B0%E6%8D%AE%E6%96%87%E7%AB%A0/pos_Brown-peel_Purple-peel-quant-identification/enrich.htm#gene15) | 4 | 68 | 0.01385098 | map00960 |
| 16 | [Zeatin biosynthesis](D:/Prof%20Gao/%E9%BB%84%E7%9A%AE%E6%95%B0%E6%8D%AE%E6%96%87%E7%AB%A0/pos_Brown-peel_Purple-peel-quant-identification/enrich.htm#gene16) | 3 | 39 | 0.01589842 | map00908 |
| 17 | [Biosynthesis of unsaturated fatty acids](D:/Prof%20Gao/%E9%BB%84%E7%9A%AE%E6%95%B0%E6%8D%AE%E6%96%87%E7%AB%A0/pos_Brown-peel_Purple-peel-quant-identification/enrich.htm#gene17) | 4 | 74 | 0.01837912 | map01040 |
| 18 | [Arachidonic acid metabolism](D:/Prof%20Gao/%E9%BB%84%E7%9A%AE%E6%95%B0%E6%8D%AE%E6%96%87%E7%AB%A0/pos_Brown-peel_Purple-peel-quant-identification/enrich.htm#gene18) | 4 | 75 | 0.01921287 | map00590 |
| 19 | [Betalain biosynthesis](D:/Prof%20Gao/%E9%BB%84%E7%9A%AE%E6%95%B0%E6%8D%AE%E6%96%87%E7%AB%A0/pos_Brown-peel_Purple-peel-quant-identification/enrich.htm#gene19) | 2 | 24 | 0.04187856 | map00965 |

**Supplementary Table 2**: Negative metabolites in relation to fruit peel coloration

| No. | Pathway | Count | All metabolites with pathway (Count. All) | P value | Pathway.ID |
| --- | --- | --- | --- | --- | --- |
| 1 | [Flavonoid biosynthesis](file://Document1#gene5#gene1) | 13 | 74 | 8.771684e-15 | map00941 |
| 2 | [Flavone and flavonol biosynthesis](file://Document1#gene5#gene2) | 11 | 51 | 9.733468e-14 | map00944 |
| 3 | [Biosynthesis of secondary metabolites](file://Document1#gene5#gene3) | 35 | 1237 | 3.886144e-12 | map01110 |
| 4 | [Metabolic pathways](file://Document1#gene5#gene4) | 45 | 2701 | 6.944173e-08 | map01100 |
| 5 | [Phenylalanine metabolism](file://Document1#gene5#gene5) | 6 | 60 | 5.528567e-06 | map00360 |
| 6 | [Biosynthesis of amino acids](file://Document1#gene5#gene6) | 7 | 128 | 4.897507e-05 | map01230 |
| 7 | [Alanine, aspartate and glutamate metabolism](file://Document1#gene5#gene7) | 4 | 28 | 5.262774e-05 | map00250 |
| 8 | [Cysteine and methionine metabolism](file://Document1#gene5#gene8) | 5 | 63 | 0.0001062297 | map00270 |
| 9 | [Carbon metabolism](file://Document1#gene5#gene9) | 6 | 114 | 0.0002119856 | map01200 |
| 10 | [Glycine, serine and threonine metabolism](file://Document1#gene5#gene10) | 4 | 50 | 0.0005214125 | map00260 |
| 11 | [ABC transporters](file://Document1#gene5#gene11) | 6 | 137 | 0.0005682011 | map02010 |
| 12 | [Isoflavonoid biosynthesis](file://Document1#gene5#gene12) | 4 | 64 | 0.001327359 | map00943 |
| 13 | [Phenylpropanoid biosynthesis](file://Document1#gene5#gene13) | 4 | 68 | 0.001662752 | map00940 |
| 14 | [Cyanoamino acid metabolism](file://Document1#gene5#gene14) | 3 | 45 | 0.00457227 | map00460 |
| 15 | [Purine metabolism](file://Document1#gene5#gene15) | 4 | 95 | 0.005570774 | map00230 |
| 16 | [Oxidative phosphorylation](file://Document1#gene5#gene16) | 2 | 16 | 0.006178899 | map00190 |
| 17 | [Aminoacyl-tRNA biosynthesis](file://Document1#gene5#gene17) | 3 | 52 | 0.006856791 | map00970 |
| 18 | [Citrate cycle (TCA cycle)](file://Document1#gene5#gene18) | 2 | 20 | 0.009594715 | map00020 |
| 19 | [Carbon fixation in photosynthetic organisms](file://Document1#gene5#gene19) | 2 | 23 | 0.01259139 | map00710 |
| 20 | [Pantothenate and CoA biosynthesis](file://Document1#gene5#gene20) | 2 | 28 | 0.01836233 | map00770 |
| 21 | [beta-Alanine metabolism](file://Document1#gene5#gene21) | 2 | 32 | 0.02363344 | map00410 |
| 22 | [Sulfur metabolism](file://Document1#gene5#gene22) | 2 | 33 | 0.02503713 | map00920 |
| 23 | [C5-Branched dibasic acid metabolism](file://Document1#gene5#gene23) | 2 | 34 | 0.02647404 | map00660 |
| 24 | [Pentose phosphate pathway](file://Document1#gene5#gene24) | 2 | 35 | 0.0279436 | map00030 |
| 25 | [Phenylalanine, tyrosine and tryptophan biosynthesis](file://Document1#gene5#gene25) | 2 | 35 | 0.0279436 | map00400 |
| 26 | [Starch and sucrose metabolism](file://Document1#gene5#gene26) | 2 | 37 | 0.03097844 | map00500 |
| 27 | [Glutathione metabolism](file://Document1#gene5#gene27) | 2 | 38 | 0.03254262 | map00480 |
| 28 | [Galactose metabolism](file://Document1#gene5#gene28) | 2 | 46 | 0.04610824 | map00052 |
| 29 | [Histidine metabolism](file://Document1#gene5#gene29) | 2 | 47 | 0.04792778 | map00340 |
| 30 | [Propanoate metabolism](file://Document1#gene5#gene30) | 2 | 48 | 0.04977318 | map00640 |

**Supplementary Table 3**. Negative regulation level of metabolites between BP and PP

| KEGG.ID | Regulate | Metabolites name |
| --- | --- | --- |
| C00354 | Up | D-Fructose-1,6-diphosphate |
| C00043 | Up | UDP-N-acetylglucosamine |
| C00092 | Up | D-Glucose 6-phosphate |
| C00620 | Up | D-Ribose-1-phosphate |
| C00158 | Down | Citric acid |
| C00152 | Down | Asparagine |
| C00049 | Down | L-Aspartic acid |
| C00383 | Down | Malonic acid |
| C00257 | Down | Gluconic acid |
| C16353 | Up | 7-Methylxanthine |
| C21593 | Up | D-Erythronic acid |
| C01546 | Down | 2-Furoic acid |
| C00051 | Down | L-Glutathione (reduced) |
| C00109 | Down | 2-Ketobutyric acid |
| C00493 | Up | Shikimic acid |
| C00879 | Up | Mucic acid |
| C10963 | Up | Terbacil |
| C00492 | Up | Raffinose |
| C00089 | Up | Sucrose |
| C00008 | Up | Adenosine diphosphate (ADP) |
| C00430 | Up | 5-Aminolevulinic acid |
| C00042 | Down | Succinic acid |
| C01424 | Up | Gallic acid |
| C02341 | Down | trans-Aconitic acid |
| C05903 | Down | Kaempferol |
| C16979 | Down | Isomangiferin |
| C10084 | Down | Isorhamnetin |
| C06476 | Down | Prostaglandin F3α |
| C10534 | Up | Tectorigenin |
| C00774 | Up | Phloretin |
| C09931 | Down | Hematoxylin |
| D08155 | Down | Manidipine |
| C04443 | Down | 3-Methoxy-5,7,3',4'-tetrahydroxy-flavone |
| C18783 | Up | Dimefuron |
| C17670 | Down | Aurantio-obtusin |
| D00395 | Down | Troglitazone |
| C17414 | Up | Isobutyrylshikonin |
| C05334 | Down | Isosakuranetin |
| C02168 | Down | Mefenamic acid |
| C09827 | Up | Pinocembrin |
| C00021 | Up | S-Adenosylhomocysteine |
| C10028 | Up | Chrysin |
| C09765 | Down | Agnuside |
| C17784 | Up | Pectolinarigenin |
| C18083 | Down | 7-Demethylsuberosin 7 |
| C17957 | Down | Irigenin |
| C11019 | Down | Pendimethalin |
| C04540 | Down | 2-Acetamido-N-β-aspartyl-deoxyhexopyranosylamine |
| C10889 | Down | Syringaresinol |
| C18869 | Down | Naptalam |
| C10503 | Up | Medicarpin |
| C00758 | Up | Bergaptol |
| C08031 | Up | Dodecyl sulfate |
| C02538 | Up | Estrone sulfate |
| C07266 | Up | Nifedipine |
| C05366 | Up | Pinoresinol |
| D03799 | Down | Dienogest |
| C07593 | Up | Rotenone |
| C10443 | Up | Curcumin |
| C10030 | Down | Chrysosplenetin B |
| C10342 | Up | Embelin |
| C16316 | Down | 13(S)-HOTrE |
| C17616 | Down | Kukoamine B |
| C09281 | Down | Ostruthin |
| C16417 | Up | Xanthohumol |
| C11135 | Up | Androsterone glucuronide |
| C08007 | Down | Buprenorphine |
| C18218 | Up | 16-Hydroxyhexadecanoic acid |
| C12276 | Down | Calcium pantothenate |
| C12136 | Up | Epigallocatechin |
| C03479 | Down | Leucovorin |
| C13708 | Up | 3-Isobutyl-1-methylxanthine (IBMX) |
| C01717 | Down | Kynurenic acid |
| C01512 | Down | Loganic acid |
| C06337 | Down | Terephthalic acid |
| C18079 | Up | Isoscopoletin |
| C03093 | Up | 7,8-Dihydroxycoumarin |
| C02906 | Down | Dihydromyricetin |
| C08174 | Up | Dexamethasone acetate |
| C01197 | Down | Caffeic acid |
| C12656 | Up | Paramethasone acetate |
| C00601 | Down | Phenylacetaldehyde |
| C00482 | Down | Sinapic acid |
| C17590 | Up | Catechin |
| C10105 | Down | Morin |
| C00097 | Down | L-Cysteine |
| C17480 | Down | Isofraxidin |
| C10715 | Down | Paeonolide |
| C20414 | Up | 4-Hydroxycoumarin |
| C01944 | Down | Octanoyl-coenzyme A |
| C02470 | Down | Xanthurenic acid |
| C01714 | Up | Isovitexin |
| C03519 | Up | N-Acetyl-L-phenylalanine |
| C01433 | Up | Loganin |
| C01541 | Up | Warfarin |
| C12621 | Down | 3-Coumaric acid |
| C09309 | Up | Rutarin |
| C10107 | Down | Myricetin |
| C09732 | Down | Eriocitrin |
| C01481 | Up | Caffeic acid |
| C10474 | Down | Lusitanicoside |
| C09793 | Up | Narirutin |
| C10108 | Down | Myricitrin |
| C10275 | Up | Polydatin |
| C01494 | Up | trans-ferulic acid |
| C01617 | Down | Taxifolin |
| C00633 | Down | 4-Hydroxybenzaldehyde |
| C14309 | Down | 5,6,7,8-Tetrahydro-2-naphthol |
| C10195 | Down | 5,7-Dihydroxy-2-(4-hydroxyphenyl)-6,8-bis[3,4,5-trihydroxy-6-(hydroxymethyl)tetrahydro-2H-pyran-2-yl]-4H-chromen-4-one |

**Table 4**. Positive regulation level of metabolites between BP and PP

| KEGG.ID | Regulate | Name |
| --- | --- | --- |
| C00315 | Down | Spermidine |
| C00750 | Down | Spermine |
| C19036 | Down | Thionazin |
| C03793 | Down | N6,N6,N6-Trimethyl-L-lysine |
| C13061 | Down | N4-Acetylsulfamethoxazole |
| C11584 | Up | 4-Methylumbelliferyl glucuronide |
| C18979 | Up | Demeton-S-methyl sulfone |
| C07589 | Down | Celecoxib |
| C00575 | Down | Adenosine cyclophosphate |
| C00407 | Down | L-Isoleucine |
| C00072 | Down | Ascorbic acid |
| C08434 | Down | N6-Methyladenine |
| C00270 | Down | N-Acetylneuraminic acid |
| C00355 | Down | L-Dopa |
| C00051 | Down | L-Glutathione (reduced) |
| C10447 | Down | 3,4-Dihydroxyphenylpropionic acid |
| C20450 | Down | 2,5-Furandicarboxylic acid |
| C01879 | Down | L-Pyroglutamic acid |
| C00147 | Up | Adenine |
| C00212 | Down | Adenosine |
| C15561 | Down | N-Benzylformamide |
| C01772 | Down | 2-Hydroxycinnamic acid |
| C00082 | Down | L-Tyrosine L |
| C00242 | Down | Guanine |
| C00387 | Down | Guanosine |
| C07807 | Down | Pirbuterol |
| C09266 | Down | Fraxin |
| C00559 | Up | 2'-Deoxyadenosine |
| C20674 | Down | 7-Methylguanosine |
| C01933 | Down | L-Norleucine |
| C00643 | Up | L-5-Hydroxytryptophan 5- |
| C16754 | Down | Aflatoxin G2 |
| C08431 | Down | Cordycepin |
| C15109 | Up | Gardenin B |
| C10038 | Down | Diosmetin |
| C01864 | Up | Methoxsalen |
| C10553 | Up | 4'-Demethylpodophyllotoxin 4'- |
| C08441 | Down | Tentoxin |
| C10084 | Down | Isorhamnetin |
| C19193 | Up | Auramine |
| C17002 | Down | Sedanolide |
| C05961 | Down | 6-Ketoprostaglandin F1α |
| C16915 | Down | Arctiin |
| C13800 | Up | 10-Nitrolinoleate |
| C09981 | Down | Zearalenone |
| C10112 | Up | Nobiletin |
| C18369 | Up | Camphor |
| C06174 | Down | Codeine |
| C10176 | Down | Rhamnetin |
| D04101 | Down | 2-Cyano-3,3-diphenylacrylic acid ethyl ester |
| C06533 | Down | Papaverine HCl |
| C09920 | Up | Brazilin |
| C07609 | Up | Parthenolide |
| C08604 | Down | Kuromanin |
| C10030 | Up | Chrysosplenetin B |
| C14484 | Down | 9-Fluoroprednisolone |
| C16972 | Up | Hirsutine |
| C11134 | Down | Testosterone glucuronide |
| C07451 | Down | Moxonidine |
| C01971 | Down | D-(+)-Maltose |
| C18605 | Down | Pyriproxyfen |
| C06999 | Down | Fexofenadine |
| C05631 | Down | Eriodictyol |
| C09827 | Up | Pinocembrin |
| C20262 | Up | Zerumbone |
| C00903 | Up | Cinnamaldehyde |
| D07089 | Down | Moxaverine |
| C17957 | Down | Irigenin |
| D01647 | Down | Ethyl paraben |
| C00584 | Down | Prostaglandin E2 |
| C10941 | Up | Furalaxyl |
| C12144 | Down | 2-Amino-1,3,4-octadecanetriol |
| C07062 | Down | Ketorolac |
| C19805 | Down | Hydrolyzed fumonisin B1 |
| C07187 | Down | Venlafaxine |
| D03813 | Up | Diflucortolone pivalate |
| C07045 | Up | Hydroxyzine |
| C04654 | Down | 15-keto Prostaglandin E1 |
| C18489 | Down | Tebuconazole |
| C00449 | Up | L-Saccharopine |
| C19527 | Up | Sudan III |
| C10502 | Up | Maackiain |
| C16995 | Down | Methyl palmitate |
| C10953 | Down | Metolachlor |
| C00762 | Up | Cortisone |
| C07591 | Down | Phenacetin |
| C01226 | Down | 12-Oxo phytodienoic acid |
| C09265 | Down | Fraxetin |
| C14794 | Down | 2,3-Dinor-8-epi-prostaglandin F2α |
| C08007 | Down | Buprenorphine |
| C17885 | Down | Atractylenolide I |
| C09001 | Down | 5,7-Dihydroxychromone 5,7 |
| C01909 | Up | Desthiobiotin |
| C13772 | Down | Clofilium |
| C02134 | Down | Allocryptopine |
| C17613 | Down | Handelin |
| C09281 | Down | Ostruthin |
| C17914 | Up | Nootkatone |
| C05299 | Up | 2-Methoxyestrone |
| C17784 | Down | Pectolinarigenin |
| C14829 | Down | (+/-)12(13)-DiHOME |
| C06087 | Up | Abietic acid |
| C18459 | Down | Difenoconazole |
| C17499 | Down | Notopterol |
| C13856 | Down | 2-Arachidonoyl glycerol |
| C18561 | Down | Pyraclostrobin |
| C01530 | Down | Stearic acid |
| D07260 | Up | Formestane |

**Supplementary Table 5:** The correlation relationship between genes and metabolites involved in flavonoid biosynthesis of *Clausena lansium L. Skeels* Peels

| Gene ID | Metabolites ID | Metabolites | Regulation | Expression  BP/PP | Gene name |
| --- | --- | --- | --- | --- | --- |
| Maker00020222  Maker00005905  Maker00022711 | C00509 | Naringenin | Up | 99.17 57.75    1.176 2.276  0.12 0.883 | *Flavanone 3-hydroxylase* (F3H) |
| Maker00008161 | C05903 | Kaempferol | Down | 12.853 33.643 | Cinnamoyl CoA reductase (CCR) |
| Maker00009648 | C05631 | Eriodictyol | Up | 42.62 7.733 | *Dihydroflavonol-4-reductase* (DFR) |
| Maker00008406 | C00389 | Quercetin | Up | 0.48 4.246 | *UDP-glycosyltransferase* (UGT) |
| BGI_novel_G003249 | C10107 | Myricetin; | Up | 0 2.996 | *Flavanone-3-hydroxylase* (F3H) |
| BGI_novel_G003249 | C02906 | Dihydromyricetin | Up | 0 1.996 | *Flavonol synthase* (FLS) |
